# Cognitive Ergonomics: Addressing Decision Fatigue in Dentistry

**DOI:** 10.34172/joddd.44818

**Published:** 2026-06-30

**Authors:** Katayoun Katebi

**Affiliations:** Department of Oral and Maxillofacial Medicine, Tabriz University of Medical Sciences, Tabriz, Iran

## Introduction

 Dentistry has long been regarded as a profession grounded in technical precision, procedural expertise, and manual dexterity. While these characteristics undoubtedly define much of clinical practice, they fail to fully capture an equally important dimension of modern dentistry: its immense cognitive demands. Throughout a single day, clinicians are required to make hundreds of decisions: many small, some highly consequential, all occurring within compressed timeframes. Interpreting radiographs, selecting anesthetic approaches, determining restorative materials, sequencing treatment, managing occlusion, responding to patient anxiety, navigating consent discussions, and adjusting to unforeseen clinical challenges all require constant mental engagement. These decisions are rarely isolated events. Instead, they occur in rapid succession, often while the clinician is simultaneously managing procedures, patient communication, staff interactions, and scheduling pressures.

 Musculoskeletal disorders among dental professionals have been extensively studied, leading to important advances in clinician posture, magnification systems, seating design, and instrument positioning.^1^ These efforts have meaningfully improved practitioner longevity and physical well-being. Yet, while dentistry has become increasingly sophisticated in protecting the clinician’s body, comparatively little attention has been paid to protecting the clinician’s mind.

## Cognitive Demands in Dentistry

 As practices become more technologically dense and patient expectations continue to rise, the cognitive burden of clinical dentistry has intensified. Digital radiography, intraoral scanners, CAD/CAM workflows, practice management software, 3D printing platforms, and increasingly complex treatment planning systems have transformed care delivery. These innovations have undeniably improved efficiency and diagnostic capability, but they have also introduced new layers of information processing, attention switching, and mental load.^2^

 This is where the concept of cognitive ergonomics becomes particularly relevant. Cognitive ergonomics refers to the design of systems, workflows, and environments in ways that align with human cognitive limitations and optimize mental performance.^3^ In healthcare, this includes reducing unnecessary mental workload, improving information flow, minimizing distractions, and structuring decisions to preserve attention and judgment. One of the most important consequences of poor cognitive ergonomics is decision fatigue.

 Decision fatigue refers to the progressive deterioration in decision quality following repeated acts of judgment and self-regulation.^4^ First described in behavioral economics and cognitive psychology, the phenomenon has since been observed across multiple healthcare settings. Physicians experiencing decision fatigue, for example, have demonstrated prescribing variability, reduced adherence to preventive recommendations, and greater reliance on default or simpler decisions later in the day.^5^ Although dentistry shares many of the same cognitive pressures, discussion of decision fatigue within the profession remains surprisingly limited.

 Importantly, decision fatigue is not synonymous with burnout, nor is it simply feeling tired at the end of a long day. Rather, it reflects depletion of executive resources, affecting attention, inhibitory control, working memory, and decision consistency.^6^ In practical terms, this may manifest subtly. A clinician late in the day may narrow a differential diagnosis too quickly, default to a familiar treatment approach without fully exploring alternatives, abbreviate patient explanations, or feel less tolerant of complexity and uncertainty.^7^ None of these behaviors necessarily reflect incompetence or negligence. They are predictable human responses to sustained cognitive load.

 Dentistry may be particularly vulnerable to this phenomenon because of the unique way it combines procedural intensity with continuous decision-making. Unlike many clinical disciplines, dental practice requires simultaneous integration of fine motor precision, visuospatial reasoning, communication management, risk assessment, environmental awareness, and rapid adaptation to unexpected procedural events. A clinician may move within minutes from diagnosing subtle radiographic pathology to performing technically demanding restorative procedures while also reassuring an anxious patient and responding to workflow interruptions.

## Clinical Implications

 Interruptions further compound the problem. Staff questions, software alerts, equipment issues, schedule disruptions, and patient-related unforeseen events fragment attention and impose repeated switching costs. Even brief disruptions can leave residual cognitive load, making re-engagement more effortful and increasing the likelihood of error. As decision quality declines, diagnostic reasoning may become less thorough, procedural consistency may erode, and patient communication may become increasingly transactional. Clinicians experiencing cognitive depletion may be more likely to omit verification steps, accept suboptimal technical outcomes, or shorten treatment discussions. These effects have implications not only for patient safety and treatment quality but also for practitioner well-being.

 Chronic exposure to high decisional density likely contributes meaningfully to professional burnout. Decision fatigue and burnout appear to exist in a reciprocal relationship: cognitive overload accelerates emotional exhaustion, while burnout further reduces cognitive resilience and tolerance for complexity.^9^ Therefore, dental systems should be intentionally designed to reduce unnecessary cognitive burden. Clinical protocols, checklists, decision trees, and evidence-based pathways reduce repetitive low-value decisions while preserving mental resources for higher-order clinical judgment. Such tools should not be perceived as constraints on expertise, but as cognitive safeguards that support it.

 Environmental design also matters. Clinical spaces and digital systems should prioritize intuitive workflows, predictable organization, visual simplicity, and minimal informational clutter. Poorly designed software, disorganized workspaces, and excessive sensory input all create avoidable cognitive friction.

 Scheduling practices warrant reconsideration as well. Complex procedures requiring maximal concentration may be better allocated to periods of peak alertness, while routine tasks and administrative activities can be strategically scheduled during lower-energy periods. Brief intervals between cognitively demanding cases may further support mental recovery and reduce attentional residue. Finally, team design plays an important role. Clear delegation protocols and optimized use of auxiliaries reduce the number of operational micro-decisions, allowing clinicians to direct cognitive resources toward diagnosis, treatment planning, and patient-centered care.

## Future Directions

 Future research in dentistry should more explicitly examine cognitive load as a determinant of clinical performance. Areas of particular interest include quantifying decision density across specialties, evaluating temporal variation in diagnostic accuracy, examining associations between workflow design and cognitive error, and developing digital tools informed by human factors principles. Artificial intelligence may eventually offer meaningful support through documentation automation, anomaly detection, and clinical decision assistance.

## Conclusion

 As dentistry continues to evolve in complexity, preserving clinician cognition will become increasingly central to patient safety, practitioner sustainability, and quality of care. The profession has spent decades refining how dentists work physically. The next step is understanding how they work mentally.

## Competing Interests

 The author is the Assistant Editor of the Journal of Dental Research, Dental Clinics, Dental Prospects (JODDD). The author declares no other competing interests concerning authorship and/or publication of this article.

## Ethical Approval

 Not applicable.
